# Enhancing the Thermo-Mechanical Property of Polymer by Weaving and Mixing High Length–Diameter Ratio Filler

**DOI:** 10.3390/polym12061255

**Published:** 2020-05-30

**Authors:** Bo Zhang, Yunmin Liang, Biwei Liu, Wei Liu, Zhichun Liu

**Affiliations:** School of Energy and Power Engineering, Huazhong University of Science and Technology (HUST), Wuhan 430074, China; M201871166@hust.edu.cn (B.Z.); ymliang@hust.edu.cn (Y.L.); M201971072@hust.edu.cn (B.L.); W_liu@hust.edu.cn (W.L.)

**Keywords:** thermo-mechanical property, polymer morphology, phonons, molecular dynamics simulation, weaving, mixing, length-diameter ratio

## Abstract

Improving thermo-mechanical characteristics of polymers can efficiently promote their applications in heat exchangers and thermal management. However, a feasible way to enhance the thermo-mechanical property of bulk polymers at low filler content still remains to be explored. Here, we propose mixing high length-diameter ratio filler such as carbon nanotube (CNT), boron nitride (BN) nanotube, and copper (Cu) nanowire, in the woven polymer matrix to meet the purpose. Through molecular dynamics (MD) simulation, the thermal properties of three woven polymers including woven polyethylene (PE), woven poly (p-phenylene) (PPP), and woven polyacetylene (PA) are investigated. Besides, using woven PE as a polymer matrix, three polymer nanocomposites, namely PE-CNT, PE-BN, and PE-Cu, are constructed by mixing CNT, BN nanotube, and Cu nanowire respectively, whose thermo-mechanical characteristics are compared via MD simulation. Morphology and phonons spectra analysis are conducted to reveal the underlying mechanisms. Furthermore, impacts of electron-phonon coupling and electrical field on the thermal conductivity of PE-Cu are uncovered via two temperature model MD simulation. Classical theoretical models are modified to predict the effects of filler and matrix on the thermal conductivity of polymer nanocomposites. This work can provide useful guidelines for designing thermally conductive bulk polymers and polymer nanocomposites.

## 1. Introduction

Polymers have been ubiquitously used in daily life and industry from water cup and plastic tube to energy storage and conversion devices due to numerous advantages like low mass density, low cost, chemical inertness, electrically insulated, and easy of processing [1,2,3]. However, the extremely low thermal conductivity of polymers (0.1~0.5 W m^−1^ K^−1^) owing to the disordered structure, chaotic polymer chain arrangement, impurities, voids, and chain entanglements limits their applications in heat exchangers, heat dissipation, and thermal management [4,5,6]. However, the thermal conductivity of single PE chain can be up to 350 W m^−1^ K^−1^ and even higher [7,8], which stimulates the interest in studying heat conduction in polymers.

Over the past decades, numerous simulations and experiments have been dedicated to investigating thermal transport in polymers from one dimensional polymer chain to three dimensional bulk polymers. Shen et al. fabricated ultra-drawn PE nanofibers with extremely high thermal conductivity [9], and they later fabricated crystalline PE nanofibers with both high strength and thermal conductivity at low temperature via local heating method [10]. Recently, Shrestha et al. fabricated PE nanofibers with room-temperature thermal conductivity of 50 W m^−1^ K^−1^ via two-step drawing method [11]. Zhang et al. found that hard Cu nanowire is more useful than graphene to improve thermal conductivity of silicone [12]. Xu et al. fabricated poly(3-hexylthiophene) films with a room-temperature thermal conductivity of 2.2 W m^−1^ K^−1^ by oxidative chemical vapor deposition [13], and they also prepared PE films with a high thermal conductivity of 62 W m^−1^ K^−1^ via flow extruding and drawing [14]. Li et al. prepared different electrospun nanofibers with thermal conductivity ranging from 0.5 to 9.3 W m^−1^ K^−1^ [15,16]. Cao et al. prepared thermally conductive PE nanowire arrays via nanoporous template wetting technique [17]. Through transient grating spectroscopy, Robbins et al. observed the phonon mean free path of semicrystalline PE can be up to 200 nm [18]. 

In addition to experiments, numerical simulations emerge as powerful tools to investigate the mechanisms of thermal transport from a microscopic perspective [19,20]. Through MD simulation, Tu et al. found that torsion and tension can be combined to enhance the thermal conductivity of PE strands [21]. Liao et al. found that the thermal conductivity of aligned PE-CNT nanocomposites can reach 60 W m^−1^ K^−1^ [22], and the heavy atoms can impede phonons transport along PE chain [23]. Ma et al. demonstrated that the redundant side chains [24] and chain rotation [25] can reduce phonon mean free path. They also developed a theoretical model related to bond energy, backbone rotation, in-plane bond ratio, and atomic amass to predict thermal conductivity of crystalline polymers [26]. Ruscher et al. uncovered the link between elasticity and thermal conductivity of several polymers [27]. An et al. investigated the effects of water content and cross-linking on the thermo-mechanical property of polyacrylamide hydrogels [28]. Zhou and Volz et al. investigated the impacts of pressure on thermal conductivity of two small organic materials [29]. Luo et al. demonstrated that strong chain backbone [30,31] and large spatial extension [32,33] play a pivotal role in thermal transport of polymers. By first-principles calculation, Shulumba et al. predicted the room-temperature thermal conductivity of polymer crystals can be over 100 W m^−1^ K^−1^ [34,35]. Yang et al. proposed using parallel-linking method to construct polymers [36] and investigated thermal transport in organic semiconductors [37]. Recently, they recommended using machine learning algorithms to search for materials with desired properties [38].

However, preparing polymer crystals needs sophisticated techniques, and incorporating excessive fillers can increase the cost and damage the intrinsic advantages of polymers. Thus, it is necessary to develop simple and feasible methods to enhance the thermal conductivity of polymer matrix and using a small quantity of fillers to fabricate thermally conductive polymer nanocomposites. Manipulating single polymer chain and elucidating structure-property relationships at molecular scale are still very challenging in experiments. Accurately characterizing experimental samples crucially depends on expensive instruments like Raman spectroscopy, scanning electron microscope, and transmission electron microscope. Besides, a high vacuum environment is indispensable for measuring thermal conductivity of nanomaterials due to the influence of convection and radiation. The experimental details including heating rate and measurement noise can induce large uncertainty. In contrast, MD simulation can easily capture the atomic vibrational details, record the atomic trajectory, calculate desired physical quantity, and provide critical insights into understanding thermal transport of polymers and polymer nanocomposites at molecular level. Hence MD simulation is adopted to investigate thermo-mechanical characteristics of polymers and polymer nanocomposites. Previous studies often focus on thermo-mechanical property of pure polymers and fabricate polymer nanocomposites via mixing excessive fillers. The crucial elements affecting thermo-mechanical property of polymer nanocomposites at low filler content and the fundamental understanding at the molecular level remain to be explored. The goal of this work is 3-fold: (1) to show a feasible method to construct polymer matrix and pinpoint the crucial factors affecting thermal conductivity of constructed polymer matrix, (2) to unravel the crucial factors influencing thermo-mechanical property of polymer nanocomposites at low filler content, (3) to uncover the underlying mechanisms from the atomistic vibration, polymer morphology, and phonons transport perspectives.

In this work, we investigate the influential factors to the thermal conductivity of woven PE, woven PPP, and woven PA via equilibrium molecular dynamics (EMD) simulation. Then, the thermo-mechanical characteristics of PE-CNT, PE-BN, and PE-Cu are explored via non-equilibrium molecular dynamics (NEMD) simulation. The impacts of electron–phonon coupling and electrical field on the thermal conductivity of PE-Cu are investigated via two temperature model molecular dynamics (TTM-MD) simulation. The underlying mechanisms are unraveled from polymer conformation, mechanical modulus, and atomic vibration perspectives. 

## 2. Model and Methodology

### 2.1. Force Field

With respect to woven polymers, the consistent valence force field (CVFF) is used to describe the interatomic interaction of woven polymers, which can be written as [39,40]

(1) $$U_{all}=\underset{b}{\sum }K_{b}{\left(r-r_{0}\right)}^{2}+\underset{\theta }{\sum }K_{\theta }{\left(\theta -\theta_{0}\right)}^{2}+\underset{\emptyset }{\sum }K_{\emptyset }\left(1+dcos\left(n\emptyset \right)\right)+\underset{i>j}{\sum }4\varepsilon \left[{\left(\frac{\sigma }{r_{ij}}\right)}^{12}-{\left(\frac{\sigma }{r_{ij}}\right)}^{6}\right]+\frac{1}{2}\overset{N}{\underset{i=1}{\sum }}\overset{N}{\underset{j=1,j\neq i}{\sum }}\frac{q_{i}q_{j}}{\varepsilon_{0}r_{ij}}$$

where $U_{all}$ denotes the total potential energy of the system; the right terms represent bond, angle, dihedral, vdW, and Coulomb interaction, respectively. The Lorentz–Berthelot mixing rules are used to specify the LJ parameters across different atomic species. (i.e., $\varepsilon_{ij}=\sqrt{\varepsilon_{i}\varepsilon_{j}},\;\;\sigma_{ij}=\left(\sigma_{i}+\sigma_{j}\right)/2$). The cutoff distance for the nonbonding interaction is set as 10 Å. Tersoff potential is used to describe the interatomic interaction of CNT and BN, and the embedded atom method (EAM) is used for interatomic interaction of Cu.

Tersoff potential can accurately capture the atomic vibrational characteristics of CNT and BN, which can be written as [41]

(2) $$U_{ij}=f_{c}\left(r_{ij}\right)\left[f_{R}\left(r_{ij}\right)+b_{ij}f_{A}\left(r_{ij}\right)\right]$$

where $U_{ij}$ is the bond energy; $f_{c}$ is a smooth cutoff function; $b_{ij}$ is the bond-order function; $f_{R}$ and $f_{A}$ stand for the repulsive and attractive potential, respectively. The EAM potential has been successfully used to describe the thermal transport of copper [42], which can be written as [43]

(3) $$U_{i}=\frac{1}{2}\underset{j\neq i}{\sum }\phi \left(r_{ij}\right)+F\left(\rho_{h,i}\right)$$

where $U_{i}$ is the site potential energy; $\phi \left(r_{ij}\right)$ is a pairwise potential between atom *i* and *j* separated by the distance $r_{ij}$; $F\left(\rho_{h,i}\right)$ is the embedding potential, which depends on the electron density $\rho_{h,i}$ at atom *i*.

### 2.2. Sample Preparation and Simulation Details

All of the simulations are carried out by employing the Large-scale Atomic/Molecular Massively Parallel Simulator (LAMMPS) package [44]. The time step is set as 0.25 fs for all of the simulations except that a smaller time step of 0.1 fs is chosen for simulation at 450 K. Periodic boundary conditions are applied to all three directions to eliminate size effects. Velocity–Verlet algorithm is used to integrate the equation of atomic motion. In order to consider the effects of electron–phonon coupling and electrical field on the thermal conductivity of PE-Cu, the TTM-MD approach is used [42], which depicts the coupled electronic and phononic thermal transport by two heat diffusion equations

(4) $$C_{e}\frac{\partial T_{e}}{\partial t}=\nabla \cdot \left(\kappa_{e}\nabla T_{e}\right)-G_{ep}\left(T_{e}-T_{p}\right)$$

(5) $$C_{p}\frac{\partial T_{p}}{\partial t}=\nabla \cdot \left(\kappa_{p}\nabla T_{p}\right)+G_{ep}\left(T_{e}-T_{p}\right)$$

where $C_{e}$, $T_{e}$, $\kappa_{e}$, $C_{p}$, $T_{p}$, $\kappa_{p}$ denote volumetric specific heat, temperature, thermal conductivity for electronic and phononic subsystem, respectively; $G_{ep}$ is the bulk Cu electron–phonon coupling parameter. The used electronic parameters for Cu are adopted from Ruan’s work [42], i.e., $C_{e}=5.26\times 10^{4}$ J m^−3^ K^−1^, $G_{ep}=5.5\times 10^{16}$ W m^−3^ K^−1^, and $\kappa_{e}=401$ W m^−1^ K^−1^. In practice, a Langevin thermostat is used to couple the electronic and phononic subsystems, which can be written as

(6) $$m_{i}{\dot{\upsilon }}_{i}=F_{i}\left(t\right)-\gamma_{i}\upsilon_{i}+F_{r}(t)$$

where $m_{i}$ and $\upsilon_{i}$ are the atomic mass and velocity; $F_{r}\left(t\right)$ is the random fluctuating force; $\gamma_{i}$ is the friction constant related to the electron–phonon interaction

(7) $$\gamma_{i}=m_{i}G_{ep}/\left(3n_{i}k_{B}\right)$$

where $n_{i}$ is the atomic number density and $k_{B}$ is the Boltzmann constant.

**Figure 1:**
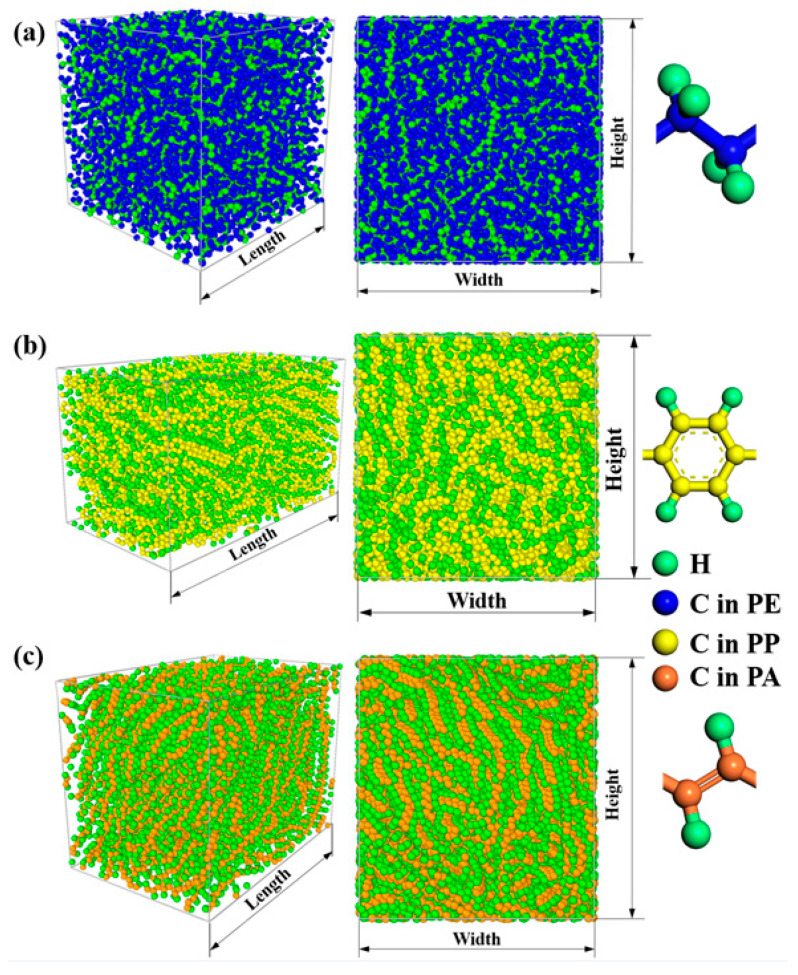
Woven polymers after relaxation in the isothermal–isobaric ensemble and the repeat unit of an individual polymer chain. (**a**) Woven PE and the ethylene unit; (**b**) Woven PPP and the phenylene unit; (**c**) Woven PA and the acetylene unit.

At first, the initial woven polymers are put in the isothermal–isobaric (NPT) ensemble for at least 1 ns to reach an equilibrium state. The target temperature and pressure are controlled at 300 K and 1 atm. During the equilibration process, the radius of gyration, volume, density, and total energy evolution is recorded and shown in Figure S1 (Supplementary Materials). It can be seen that the radius of gyration, volume, density, and total energy all reach a steady value after NPT relaxation, which implies that these three systems all reach the corresponding equilibrium states. The sizes of the equilibrium states for woven PE, woven PPP, and woven PA are 46 × 46 × 46 ${Å}^{3}$, 71 × 41 × 41 ${Å}^{3}$, and 58 × 39 × 43 ${Å}^{3}$, respectively. Figure 1 shows the structure of three woven polymers and their repeat unit. Then these woven polymers are equilibrated in the canonical (NVT) ensemble for 1 ns, whose temperatures are all controlled at 300 K using Nos$\overset{´}{e}$–Hoover thermostats [45]. After that, they run in the microcanonical (NVE) ensemble for 2 ns and the heat current is recorded at each step. The microscopic expression of heat current ***J*** is

(8) $$J=\underset{i}{\sum }\upsilon_{i}e_{i}+\frac{1}{2}\underset{i}{\sum }\underset{j\neq i}{\sum }(r_{ij}⨂F_{ij})\cdot \upsilon_{i}$$

where $e_{i}$ denotes the total energy of atom i; $r_{ij}$ and $F_{ij}$ are the relative position and interatomic force between atom i and j. Based on the fluctuation–dissipation theorem [46] and linear response theory, the thermal conductivity κ can be calculated by Green–Kubo formula

(9) $$\kappa =\frac{1}{3k_{B}VT^{2}}\int_{0}^{\tau_{c}}\langle J\left(0\right)\cdot J\left(t\right)\rangle dt$$

where *V* is the volume of the system; 〈J(0)⋅J(t)〉 is the heat current autocorrelation function (HCACF);$\;\tau_{c}$ is the upper limit of integration time, and the angular bracket denotes the time average.

In NEMD approach, the heat flux *J* can be calculated from the energy tally *Q* recorded on the heat source and sink, which can be given by

(10) $$J=\frac{1}{2S}(\left|\frac{dQ_{in}}{dt}\right|+\left|\frac{dQ_{out}}{dt}|\right)$$

where *S* is the cross-sectional area. Thermal conductivity can also be calculated according to Fourier’s law,

(11) κ=−J/∇T

Traditionally, the temperature gradient is often calculated by linear fitting the temperature distribution away from the thermostats. However, a recent study indicates that the nonlinear parts close to the thermostats should not be excluded [47]. Hence the later method is also used to compare thermal conductivity of different systems. 

**Figure 2:**
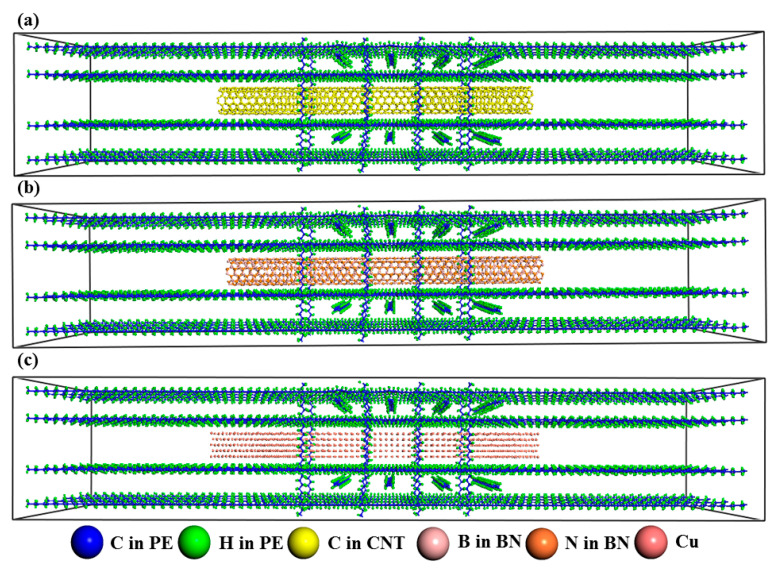
(**a**) Pristine structure of PE-CNT; (**b**) pristine structure of PE-BN nanotube; (**c**) pristine structure of PE-Cu nanowire.

The preparation process for PE-CNT, PE-BN, and PE-Cu is very similar to woven polymers. The PE matrix consists of 16 PE chains in *z* direction and 8 PE chains in *x*, *y* direction, respectively. The total number of atoms for PE is 9216, and the feature sizes for pristine Cu nanowire, CNT, and BN nanotube are 7.23 × 7.23 × 101.21 ${Å}^{3}$, 8.14 × 100 ${Å}^{2}$, and 8.14 × 100 ${Å}^{2}$. The total number of atoms for PE-CNT, PE-BN, and PE-Cu are 10176, 10176, and 9929. The initial sizes of PE-CNT, PE-BN, and PE-Cu are all 40 × 40 × 210 ${Å}^{3}$. Figure 2 displays the pristine structure of PE-CNT, PE-BN nanotube, and PE-Cu nanowire. The main view and lateral view of three polymer nanocomposites are shown in Figure S2 (Supplementary Materials). These three systems are firstly relaxed in the NPT ensemble for 10 ns and the target temperature and pressure are maintained at 300 K and 1 atm. The corresponding volume and total energy evolution are recorded and displayed in Figure S3 (Supplementary Materials). It can be seen that the volume and total energy converge to a steady value during the NPT relaxation, which denotes three systems, all reach their equilibrium states. Then the systems run in the NVT ensemble for 1 ns to reach equilibrium state. Later the thin layers (13 Å) at each end of the system are fixed to hinder the heat transfer across the boundary and translational drift of the system. Then, the systems run in the NVE ensemble. Meanwhile, the 15 Å thick layers next to the fixed layers are used as the heat source (307 K) and heat sink (293 K) with temperature controlled by Langevin thermostats. The systems run 8 ns to fully reach steady state. The final thermal conductivity is averaged over 4 independent simulations with different initial conditions.

The stress-strain (σ−ε) simulations are conducted to investigate the mechanical property of woven PE, PE-CNT, PE-BN, and PE-Cu. The simulations about mechanical characteristics are carried out in the NPT ensemble along z direction with 1 atm in the lateral directions. The strain rate is all set as 0.001/ps, and stress is calculated by

(12) $$\sigma_{\alpha \beta }=\frac{1}{V}\left(\overset{N}{\underset{i=1}{\sum }}m_{i}\upsilon_{\alpha }^{i}\upsilon_{\beta }^{i}+\frac{1}{2}\overset{N-1}{\underset{i=1}{\sum }}\overset{N}{\underset{j=i+1}{\sum }}r_{ij,\alpha }F_{ij,\beta }\right)$$

where *N* stands for the number of atoms in the system; α and β denote the components of Cartesian coordinate. The Young’s modulus can be obtained by linear fitting the stress–strain curves, which can be given by

(13) $$K=\frac{d\sigma }{d\varepsilon }{|}_{\varepsilon \to 0}$$

The spatial extension of PE chains can be evaluated by the radius of gyration (*R*_g_), which is defined as

(14) $$R_{g}^{2}=\frac{1}{M}\underset{i}{\sum }m_{i}{\left(r_{i}-r_{cm}\right)}^{2}$$

where *M*, $r_{i}$, $r_{cm}$ represent the total mass of the system, the position of atom *i*, and the center of the group, respectively. The radial distribution function (RDF) can describe the atomic distribution, which can be written as

(15) $$g\left(r\right)=\frac{n\left(r\right)}{4\pi r^{2}n\Delta r}$$

where *r* is the distance of reference atoms and neighbor atoms; n(r) is the number of atoms in a spherical shell with width Δr. Here, the carbon atoms in PE chain are chosen as reference atoms, and Δr is set as 0.2 Å. The cutoff distance for RDF calculation is the same as the cutoff distance of nonbonding interaction. The X-ray diffraction (XRD) pattern is based on a mesh of reciprocal lattice nodes defined by the entire simulation domain using a simulated radiation of wavelength lambda [48], which can characterize the degree of crystallinity of the system. 

According to the kinetic theory, the thermal conductivity is intimately associated with phonons characteristics, which can be written as

(16) $$\kappa =\frac{1}{3V}\int_{0}^{\omega_{m}}\hbar \omega \frac{\partial f_{BE}}{\partial T}\upsilon_{g}\left(\omega \right)\upsilon_{g}\left(\omega \right)\tau \left(\omega \right)\mathrm{VDOS}\left(\omega \right)d\omega$$

where $\hbar ,\omega ,\omega_{m},\;f_{BE}$ are the reduced Planck’s constant, angular frequency, upper limit of integration frequency, and Bose–Einstein distribution function; $\upsilon_{g}\left(\omega \right)$, τ(ω), and VDOS(ω) are frequency-dependent group velocity, phonon relaxation time, and vibrational density of states (VDOS), respectively. In the classical limit, the volumetric heat capacity $c_{v}$ can be estimated by Dulong–Petit‘s law

(17) $$c_{v}=\frac{1}{V}\int_{0}^{\omega_{m}}\hbar \omega \frac{\partial f_{BE}}{\partial T}\mathrm{VDOS}\left(\omega \right)d\omega \approx \frac{3Nk_{B}}{V}=3\bar{n}k_{B}$$

The VDOS spectra determines the frequency distribution of phonons and can be calculated by the Fourier transforming velocity autocorrelation function (VACF), which can be given by [49]

(18) $$\mathrm{VDOS}\left(\omega \right)=\int_{-\infty }^{+\infty }\frac{\langle \upsilon \left(0\right)\cdot \upsilon \left(t\right)\rangle }{\langle \upsilon \left(0\right)\cdot \upsilon \left(0\right)\rangle }\cos\left(\omega t\right)dt$$

where $\frac{\langle \upsilon \left(0\right)\cdot \upsilon \left(t\right)\rangle }{\langle \upsilon \left(0\right)\cdot \upsilon \left(0\right)\rangle }$ is the normalized VACF. The mode participation ratios (MPR) are calculated to characterize the localization of phonons. The smaller MPR indicates that fewer atoms participate in the eigenvibration, and thus, phonons are more localized. Regardless of polarization information of phonons, the MPR can be defined as [20]

(19) $$\mathrm{MPR}\left(\omega \right)=\frac{1}{N}\frac{{\left(\sum_{i}{\mathrm{VDOS}}_{i}{\left(\omega \right)}^{2}\right)}^{2}}{\sum_{i}{\mathrm{VDOS}}_{i}{\left(\omega \right)}^{4}}$$

where ${\mathrm{VDOS}}_{i}\left(\omega \right)$ is the local VDOS of *i*th atom. The MPR from MD simulation includes all order of anharmonic information and can reflect phonons behaviors at room temperature. Chen and Li et al. suggest using averaged MPR to consider the overall phonons localization effect [50], which can be written as

(20) $${\mathrm{MPR}}_{\mathrm{ave}}\left(\omega \right)=\int_{0}^{\omega }\mathrm{MPR}\left(\omega \right)\cdot \mathrm{VDOS}\left(\omega \right)d\omega /\int_{0}^{\omega }\mathrm{VDOS}\left(\omega \right)d\omega$$

## 3. Results and Discussion

Through EMD simulation, we compare thermal conductivity of three woven polymers and find that high bond and dihedral energy constants are beneficial to low-frequency phonons. Through NEMD simulation, we investigate thermo-mechanical characteristics of woven PE, PE-CNT, PE-BN, and PE-Cu. We find that mixing high length-diameter ratio filler plays a more effective role in fabricating thermally conductive polymer nanocomposites especially at low filler content, while the thermal conductivity of filler itself does not matter much. In addition, we investigate the role of electron–phonon coupling and electrical field intensity in thermal transport characteristic of PE-Cu nanowire via TTM-MD simulation. We find that the electrical field has a negligible effect on the thermal conductivity of weak-polar systems and electron–phonon coupling does not play a crucial role in the thermal conductivity of polymer–metal nanocomposites with a low volume of metal filler, which is a contrast to thermal transport across metal-dielectric interface [51]. Thermal conductivity decomposition analysis is conducted to unravel the underlying mechanisms. Classical Behrens and Lewis models are modified to observe the effects of filler and polymer matrix on thermal transport characteristic of polymer nanocomposites. The effects of electrical field and electron–phonon coupling on strong-polar systems with a high volume of metal filler will be explored in our next work. 

### 3.1. Thermal Conductivity Comparasion

**Figure 3:**
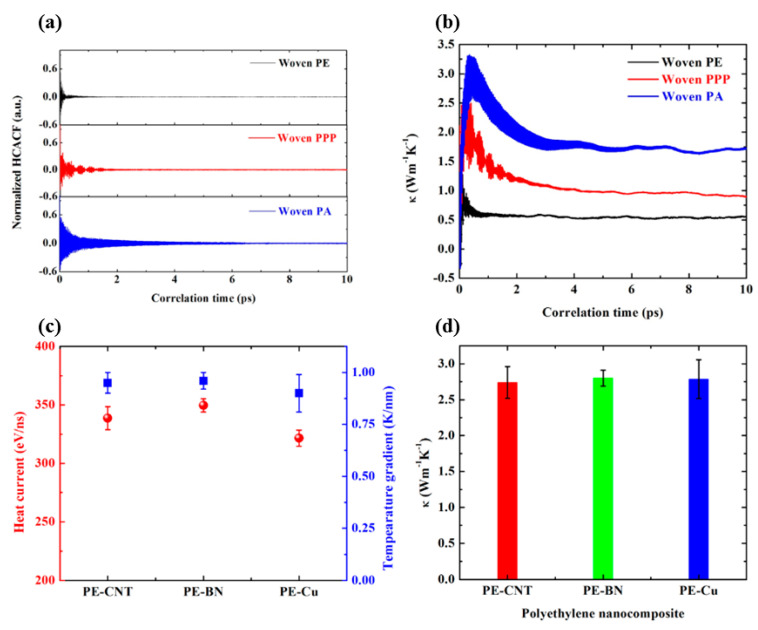
Heat current and thermal conductivity from EMD and NEMD simulation. (**a**) Normalized heat current autocorrelation function as a function of correlation time for woven PE, woven PPP, and woven PA in EMD simulation; (**b**) running thermal conductivity as a function of correlation time for woven PE, woven PPP, and woven PA in EMD simulation; (**c**) steady state heat current and temperature gradient for PE-CNT, PE-BN, and PE-Cu in NEMD simulation; (**d**) thermal conductivity of PE-CNT, PE-BN, and PE-Cu in NEMD simulation.

The normalized HCACF evolution versus correlation time for woven PE, woven PPP, and woven PA in EMD simulation is displayed in Figure 3a. The normalized HCACF decays dramatically in the first 4 ps, then fluctuates around 0 in the later 6 ps. The attenuation of normalized HCACF results from anharmonic phonon–phonon scatterings, and longer attenuation implies longer phonon lifetime [37]. Hence the phonon lifetime of woven PA is the longest, followed by woven PPP, and the last is woven PE. The running thermal conductivity in Figure 3b shows that woven PA has the highest thermal conductivity with 1.71 W m^−1^ K^−1^, followed by woven PPP, and the last is woven PE with 0.51 W m^−1^ K^−1^.

The steady state heat current and temperature gradient displayed in Figure 3c are calculated from the energy tally and linear fitting the temperature distribution away from Langevin thermostats. The steady state energy tally and temperature distribution are shown in Figure S4 (Supplementary Materials). Figure 3d compares thermal conductivity of PE-CNT, PE-BN, and PE-Cu. Although the intrinsic thermal conductivity of CNT, BN nanotube, and copper nanowire is significantly different from each other [42,52,53], the thermal conductivity of their nanocomposites is very similar. Hence, we conclude that the intrinsic thermal conductivity of filler itself does not play an important role in the thermal transport characteristic of polymer nanocomposites at low volume content.

### 3.2. Phonons Spectra and Morphology Analysis for Woven Polymers

**Figure 4:**
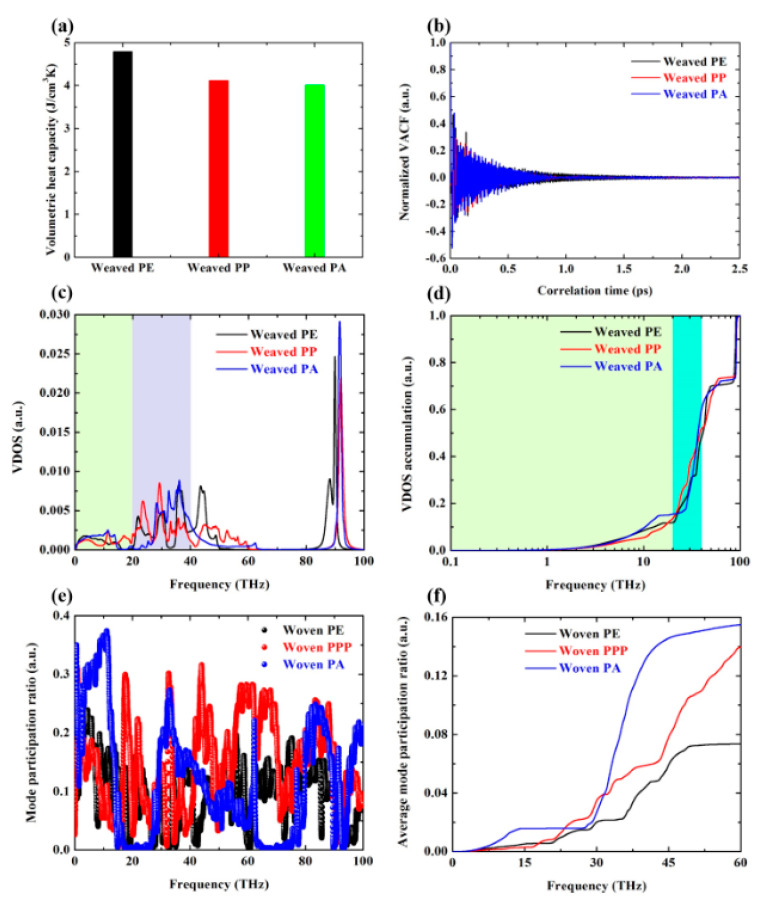
Phonons spectra analysis for woven PE, woven PPP, and woven PA. (**a**) Volumetric specific heat for woven PE, woven PPP, and woven PA; (**b**) normalized velocity autocorrelation function as a function of correlation time for woven PE, woven PPP, and woven PA; (**c**) vibrational density of states as a function of frequency for woven PE, woven PPP, and woven PA; (**d**) accumulated vibrational density of states as a function of frequency for woven PE, woven PPP, and woven PA; (**e**) mode participation ratio as a function of frequency for woven PE, woven PPP, and woven PA; (**f**) average mode participation ratio as a function of frequency for woven PE, woven PPP, and woven PA.

**Figure 5:**
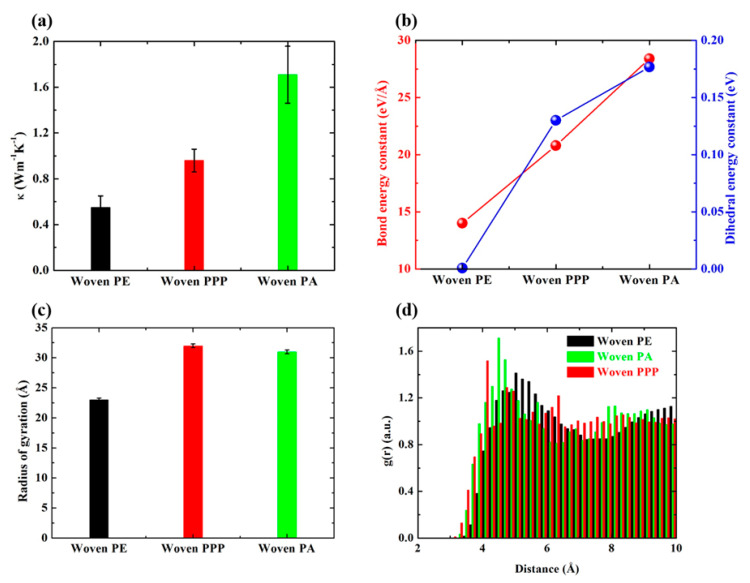
Thermal conductivity comparison and morphology analysis for three woven polymers. (**a**) Thermal conductivity for woven PE, woven PPP, and woven PA; (**b**) bond and dihedral energy constant for woven PE, woven PPP, and woven PA; (**c**) radius of gyration for woven PE, woven PPP, and woven PA; (**d**) the radial distribution function for woven PE, woven PPP, and woven PA.

In order to understand the reasons for different thermal conductivity of three woven polymers (See Figure 3b), phonons spectra analysis is carried out, and the results are displayed in Figure 4. As shown in Figure 4a, the volumetric specific heat of three woven polymers calculated by Dulong–Petit’s law is close to each other, which is not the main factor leading to different thermal conductivity. Normalized VACF shown in Figure 4b attenuates dramatically in the first 1 ps, then fluctuates around 0 in the later 1.5 ps. The corresponding VDOS spectra shown in Figure 4c indicates that the low-frequency phonons in woven PA account for a large proportion, followed by woven PPP, and the last is woven PE. The accumulated VDOS spectra shown in Figure 4d is calculated by integrating the VDOS with respect to vibrational frequency, which reflects the same feature as VDOS spectra. Shenogin et al. [54] found that most phonons are localized and make little contribution to thermal conductivity of polymers, and only a few low-frequency propagating modes play a crucial role in thermal transport of polymers. Our atomistic vibration analysis is consistent with Shenogin’s work. Figure 4e, f compares the MPR and average MPR of three woven polymers. It can be seen that woven PA has a clearly highest MPR in the range of 0~20 THz, which indicates that more atoms participate in this eigenvibration and excite more low-frequency phonons. The bond and dihedral energy constant shown in Figure 5b clearly illustrates that the woven PA has the highest bond and dihedral energy constant, and thus has the stiffest backbone in three woven polymers. The *R*_g_ shown in Figure 5c illustrates that woven PA and woven PPP have larger spatial extensions than woven PE. Compared with woven PE, the RDF shown in Figure 5d demonstrates that woven PA and woven PPP have more distinct peaks. Hence woven PA and woven PPP have a more ordered structure and a better thermal transport character. Moreover, the thermal conductivities of three woven polymers are all higher than their amorphous counterpart. Therefore, weaving polymer chain with high bond and dihedral energy constant can easily fabricate bulk polymers with improved thermal transport characteristic.

### 3.3. Phonons Spectra and Morphology Analysis for PE Nanocomposites

**Figure 6:**
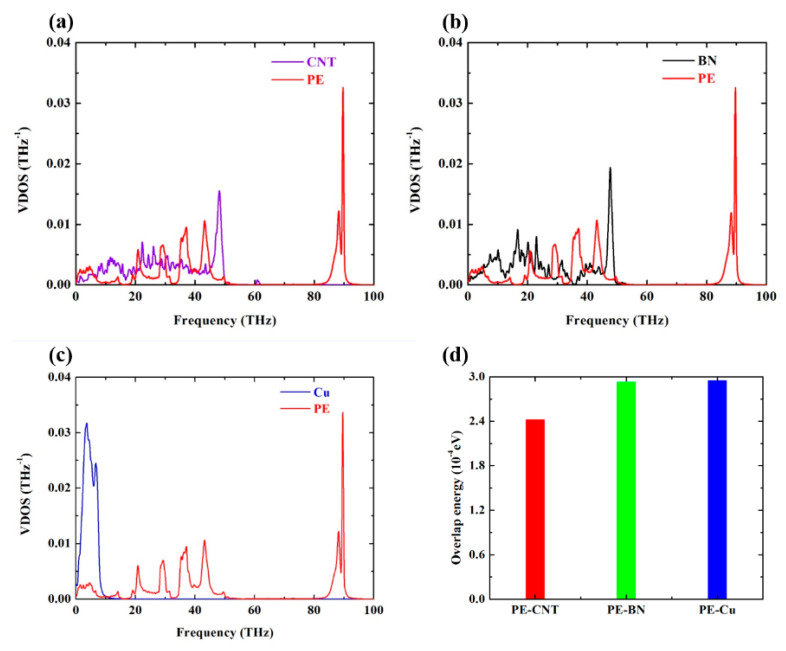
Phonons spectra and overlap energy for PE-CNT, PE-BN, and PE-Cu. (**a**) Phonons spectra as a function of frequency for PE-CNT; (**b**) phonons spectra as a function of frequency for PE-BN; (**c**) phonons spectra as a function of frequency for PE-Cu; (**d**) phonons overlap energy for PE-CNT, PE-BN, and PE-Cu.

The atomic velocities of PE matrix, CNT, BN nanotube, and Cu nanowire are extracted each step in the NVE ensemble for 2.5 ps. By applying Fourier transformation to the normalized VACF, the VDOS spectra is calculated and shown in Figure 6a–c. Zhang et al. recommends using phonons overlap energy to quantify the atomic vibrational energy transport ability [55]

(21) $$E_{overlap}=\int_{0}^{\nu }\frac{h\nu }{\exp\left(\frac{h\nu }{k_{B}T}\right)-1}\cdot g_{0}\left(\nu \right)d\nu$$

where hν is the energy for per phonon; $\frac{1}{\exp\left(\frac{h\nu }{k_{B}T}\right)-1}$ is the Bose–Einstein distribution function and $g_{0}\left(\nu \right)$ is the overlap of VDOS. Here the phonons overlap energy is used to quantify the interfacial thermal transport between PE matrix and CNT, BN, and Cu. As can be seen from Figure 6a, b, there is no VDOS overlap between PE and CNT, BN in the frequency range from 50 to 100 THz. Thus only low- and middle-frequency phonons can transport across the interface between PE and CNT, BN. For PE and Cu, the frequency range of VDOS overlap is from 0 to 20 THz, which denotes that only low-frequency phonons have the ability transport across the interface between PE and Cu. Although the overlap frequency range for PE-Cu is narrower than PE-CNT and PE-BN, the proportion of low-frequency phonons in PE-Cu is larger than PE-CNT and PE-BN. The phonons overlap energy shown in Figure 6d indicates that PE-Cu and PE-BN have better interfacial thermal transport ability. However, one thing should be noticed that the order of phonons overlap energy is at the $10^{-4}$ eV, which is so weak that the superior thermal transport ability of filler cannot make a difference to the overall thermal conductivity of PE nanocomposites. Therefore, we argue that the intrinsic thermal conductivity of filler plays a negligible role in improving the overall thermal conductivity of polymer nanocomposites at low filler content. 

**Figure 7:**
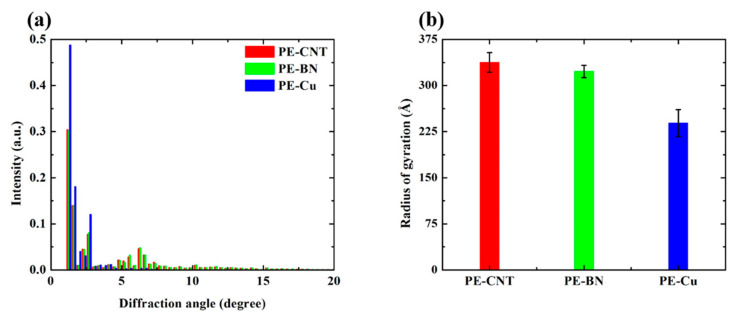
(**a**) X-ray diffraction pattern for PE-CNT, PE-BN, and PE-Cu; (**b**) radius of gyration for PE-CNT, PE-BN, and PE-Cu.

The XRD pattern shown in Figure 7 illustrates the crystallinity of PE-CNT, PE-BN, and PE-Cu. It can be seen that PE-Cu has the highest peaks at a small diffraction angle, which implies that PE-Cu has a higher crystallinity than PE-CNT and PE-BN. The similar XRD peak for PE-CNT and PE-BN implies that the crystallinity of PE-CNT and PE-BN is close. Figure 7b depicts the *R*_g_ of PE-CNT, PE-BN, and PE-Cu. Although the crystallinity of PE-CNT and PE-BN is lower than PE-Cu, their R_g_ is larger than PE-Cu. Hence the overall thermal transport performance of PE-CNT, PE-BN, and PE-Cu is similar. 

### 3.4. Temperature-Dependent Thermal Conductivity of PE-CNT

**Figure 8:**
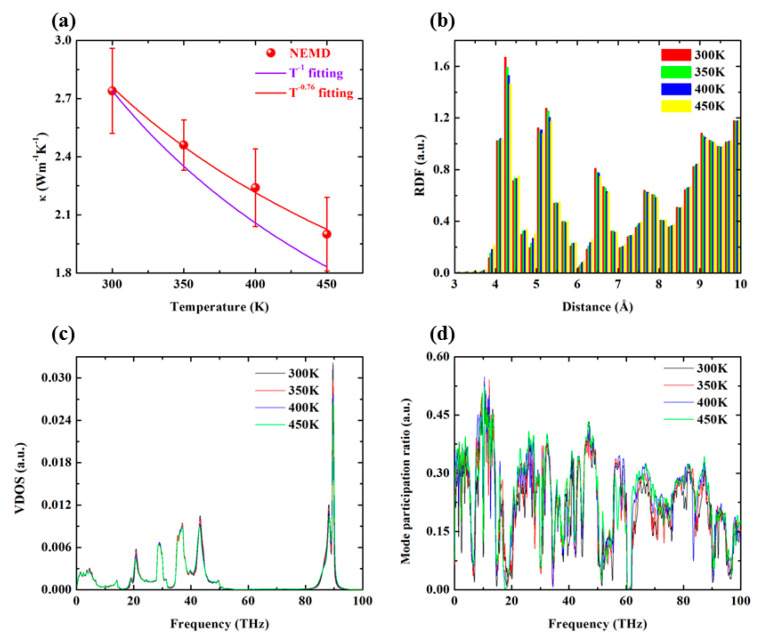
Effects of temperature on thermal transport characteristic of PE-CNT. (**a**) Thermal conductivity of PE-CNT as a function of temperature; (**b**) the radial distribution function of PE-CNT versus temperature; (**c**) vibrational density of states for PE-CNT versus temperature; (**d**) mode participation ratio of PE-CNT versus temperature.

Through NEMD simulation, the thermal conductivity of PE-CNT is calculated at different temperature, and the results are shown in Figure 8a. It can be seen that the thermal conductivity of PE-CNT is clearly deviated from classical T^−1^ law, while T^−0.76^ has a better fit. 

Figure 8b depicts the temperature-dependent RDF, which shows that the ordered degree of PE-CNT decreases slightly with the increase of temperature. The VDOS spectra shown in Figure 8c illustrates that more high-frequency phonons are excited at high temperature and thus induce more phonon–phonon scatterings, which is detrimental to thermal transport of PE-CNT. The MPR spectra shown in Figure 8d illustrate that the MPR slightly increases with the increase of temperature. 

### 3.5. Electron–Phonon Coupling and Elecrtical Field

**Figure 9:**
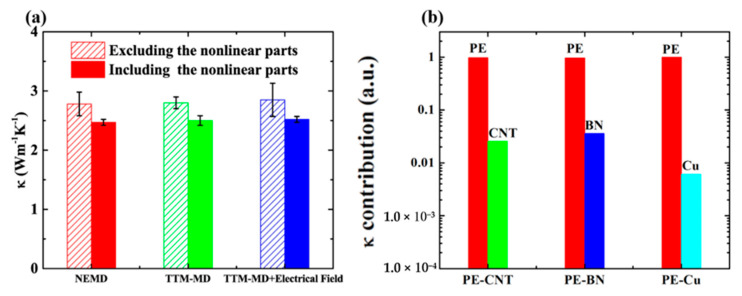
(**a**) Influences of electron–phonon coupling, electrical field, and thermostats on the thermal conductivity of PE-Cu; (**b**) thermal conductivity decomposition for PE-CNT, PE-BN, and PE-Cu.

Ruan et al. [42,56] found that whether including electron–phonon coupling in MD simulation has a significant influence on interfacial thermal conductance between metal–dielectric interface. However, the effects of electron–phonon coupling on the thermal conductivity of metal–polymer nanocomposites remain to be explored. Here the thermal conductivity of PE-Cu is calculated via TTM-MD simulation, and the results are compared with general NEMD simulation. Furthermore, an electrical field with 0.5 $V\;{Å}^{-1}$ along temperature gradient direction is applied to PE-Cu, and the thermal conductivity is also calculated. The effects of nonlinear parts in the temperature distribution close to thermostats on the thermal conductivity of PE-Cu are investigated, and the results are illustrated in Figure 9a. In contrast to interfacial thermal transport, the influences of electron–phonon coupling on thermal conductivity of PE-Cu can be negligible, which is attributed to the low volume content of Cu. By decomposing total heat current of PE nanocomposites into PE matrix and filler, the individual contribution to total thermal conductivity from PE and filler is revealed. As shown in Figure 9b, the thermal conductivity contribution from PE matrix can account for 90%. Hence the electron–phonon coupling in Cu does not influence thermal conductivity of PE-Cu. Similarly, the intrinsic thermal conductivities of CNT, BN, and Cu are trivial to overall thermal conductivity of their polymer nanocomposites. In addition, the nonlinear temperature distribution close to the thermostats can influence the absolute value of thermal conductivity but produce negligible influences on final conclusion. The contribution of electron–phonon coupling to thermal conductivity of polymer–metal nanocomposites at relatively high filler content will be investigated in our next work. 

### 3.6. Mechanical Modulus Comparison

**Figure 10:**
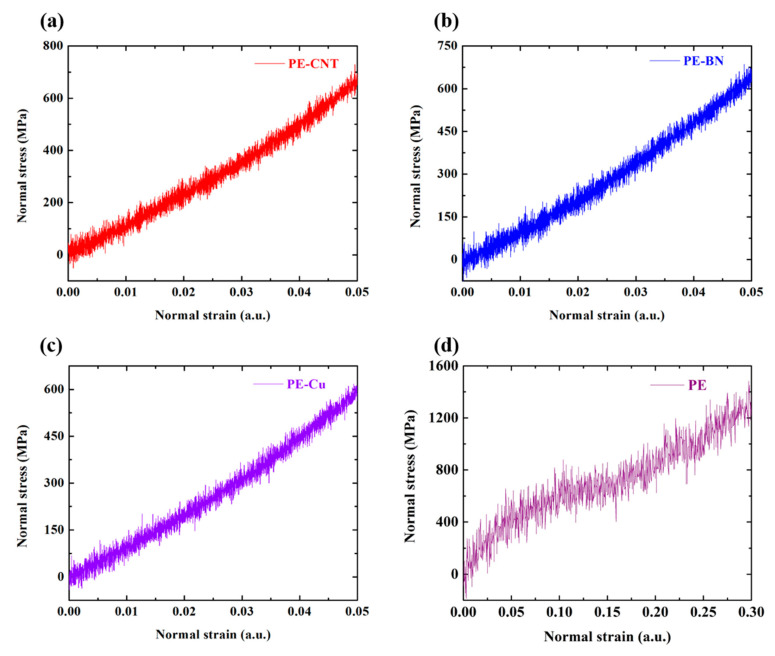
Stress–strain relations. (**a**) Normal stress versus normal strain for PE-CNT; (**b**) normal stress versus normal strain for PE-BN; (**c**) normal stress versus normal strain for PE-Cu; (**d**) normal stress versus normal strain for woven PE.

**Figure 11:**
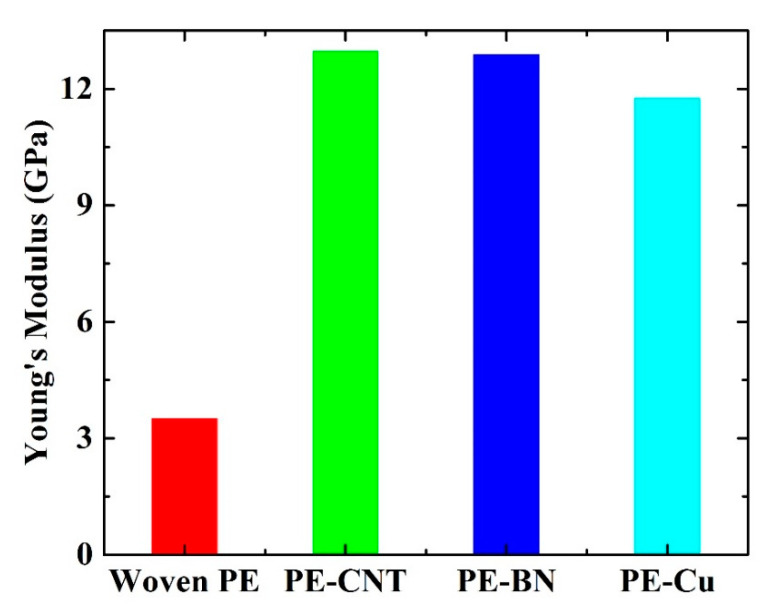
Young’s modulus of woven PE, PE-CNT, PE-BN, and PE-Cu.

In real application, the mechanical modulus of polymer nanocomposites is an essential parameter. Hence the stress-strain simulation is performed in the NPT ensemble along *z* direction. Figure 10 shows the normal stress versus normal strain for PE-CNT, PE-BN, PE-Cu, and woven PE. By linear fitting the stress–strain curves, the Young’s modulus of these systems can be obtained, and the results are shown in Figure 11. It can be seen that the mechanical modulus of PE-CNT and PE-BN are extremely close, while the mechanical modulus of CNT [57] and BN nanotube [58] is different. It is the similar morphology of CNT and BN nanotube that results in this phenomenon. In contrast, the mechanical modulus of PE-Cu is lower than that of PE-CNT and PE-BN. Hence the intrinsic morphology of filler is more important than the intrinsic mechanical modulus for fabricating stiff polymer nanocomposites. 

### 3.7. Theoretical Model Prediction

**Figure 12:**
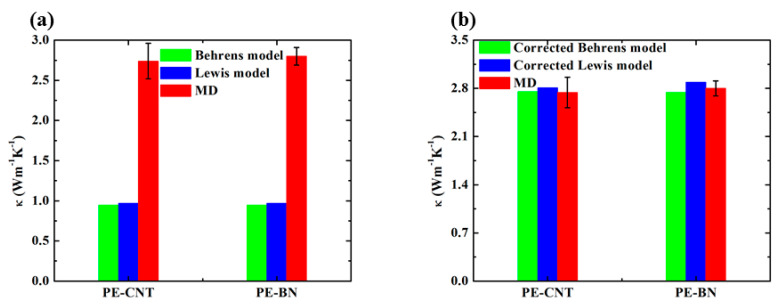
(**a**) Thermal conductivity of PE-CNT and PE-BN calculated from theoretical models and NEMD simulation; (**b**) thermal conductivity of PE-CNT and PE-BN calculated from corrected theoretical models and NEMD simulation.

Mechanical modulus is directly related to sound speed of material. For low-frequency phonons close to Γ point, the phonon group velocity can be approximated by sound speed [59]. Classical theoretical models often concentrate on thermal conductivity of filler and polymer matrix but pay little attention to mechanical modulus of systems. Here we propose to add a term η related to mechanical modulus to classical Behrens and Lewis models [60]

(22) $$\eta =\frac{K_{\mathrm{PE}-\mathrm{fillers}}}{K_{\mathrm{PE}}}+\zeta$$

(23) $$\kappa_{\mathrm{PE}-\mathrm{fillers}}^{B-C}=\eta \kappa_{\mathrm{PE}}\left[\left(p/\eta +1\right)+\left(p/\eta -1\right)f\right]/\left[\left(p/\eta +1\right)-\left(p/\eta -1\right)f\right]$$

(24) $$\kappa_{\mathrm{PE}-\mathrm{fillers}}^{L-C}=\eta \kappa_{\mathrm{PE}}\left(1+Af\left(p/\eta -1\right)/\left(p/\eta +A\right)\right)/\left(1-Af\chi \right)$$

where $K_{\mathrm{PE}-\mathrm{fillers}}$, $K_{\mathrm{PE}}$ are Young’s modulus of polymer nanocomposite and PE matrix; ζ is a fitted constant to consider remanent factors except mechanical modulus; *f* is the volume content of fillers and $p=\kappa_{fillers}/\kappa_{PE}$; B=(p−1)/(p+A), $\chi =1+\left(1-\psi_{m}\right)f/\psi_{m}^{2}$, A and $\psi_{m}$ are empirical constants. Through NEMD simulation, the thermal conductivity of PE matrix, CNT, and BN nanotube are 0.9 W m^−1^ K^−1^, 195.85 W m^−1^ K^−1^, and 105.32 W m^−1^ K^−1^, respectively. Figure 12 compares the thermal conductivity of PE-CNT and PE-BN from NEMD simulation and theoretical models. It can be seen that classical theoretical models underestimate thermal conductivity of PE-CNT and PE-BN due to the unique structure and morphology. In contrast, the thermal conductivity predicted by the corrected Berens and Lewis models is consistent with NEMD simulation.

## 4. Conclusions

To summarize, we propose constructing thermally conductive polymers and polymer matrix via weaving polymer chain. Through EMD simulation, we find that polymer chain with high bond and dihedral energy constant is more suitable to construct polymers with high thermal conductivity. Phonons spectra analysis indicates that low-frequency phonons in polymer chain with high bond and dihedral energy constant account for a larger proportion. Radius of gyration and radial distribution function comparison indicate that weaving method is beneficial to forming ordered structure and thus reduces phonon scatterings. Using woven PE as a matrix, PE-CNT, PE-BN, and PE-Cu are constructed via mixing CNT, BN nanotube, and Cu nanowire. Through NEMD simulation, thermal conductivity of these polymer nanocomposites is calculated. Thermal conductivity analysis demonstrates the contribution to total thermal conductivity from PE matrix can be up to 90%. Atomistic vibration and phonons overlap energy analysis confirm that only low-frequency phonons can transport across the interface and make a contribution to overall thermal transport characteristic. XRD and radius of gyration analysis demonstrate that it is the morphology of filler, instead of the intrinsic thermal conductivity, that plays a crucial role in tailoring thermal conductivity of polymer nanocomposites at low filler content. Besides, we find that the thermal conductivity of PE-CNT variation with temperature is clearly deviated from classical T^−1^ law. Radial distribution function and phonons spectra analysis illustrate that the ordered degree of PE-CNT decreases with the increase of temperature and more high-frequency phonons are excited, which induces more phonon scatterings. By conducting stress–strain simulation, we find that PE-CNT and PE-BN have nearly similar mechanical modulus due to the similar cylindrical structure of CNT and BN nanotube. Mixing filler with high length–diameter ratio can construct polymer nanocomposites with higher mechanical modulus. Classical Behrens and Lewis models are modified by considering mechanical modulus change to predict thermal conductivity of PE-CNT and PE-BN due to the unique nanostructure. Furthermore, through TTM-MD simulation, we find that electron–phonon coupling and electrical field have negligible influences on thermal conductivity of PE-Cu system due to the extremely low volume content of Cu nanowire, which is a sharp contrast to thermal transport across the metal–dielectric interface. The effects of electron–phonon coupling at high volume content will be investigated in our next work. 

## Supplementary Materials

The following is available online at https://www.mdpi.com/2073-4360/12/6/1255/s1. Figure S1: Typical physical property evolution of woven PE, woven PPP, and woven PA in the isothermal−isobaric (NPT) ensemble. Figure S2: The pristine structure of PE-CNT, PE-BN, and PE-Cu. Figure S3: Typical physical property evolution of PE-CNT, PE-BN, and PE-Cu in the isothermal−isobaric (NPT) ensemble. Figure S4: Steady state energy tally and temperature distribution in non-equilibrium molecular dynamics simulation for PE-CNT, PE-BN, and PE-Cu.
