# Supplementary material for: Enhancing the Thermo-Mechanical Property of Polymer by Weaving and Mixing High Length–Diameter Ratio Filler
Source: Polymers (Basel). 2020 May 30;12(6):1255. doi: 10.3390/polym12061255 (PMC7361691; doi:10.3390/polym12061255)
Supplement: Supplementary file 1 [file polymers-12-01255-s001.pdf]

Supporting information

Enhancing the Thermo-mechanical Property of Polymer by Weaving and Mixing High Length-diameter Ratio Filler

Bo Zhang, Yunmin Liang, Biwei Liu, Wei Liu, Zhichun Liu*

School of Energy and Power Engineering, Huazhong University of Science and Technology (HUST), Wuhan 430074, China

*Corresponding Authors. E-mail: zcliu@hust.edu.cn (Zhichun Liu), Tel: 86-27-87542618.

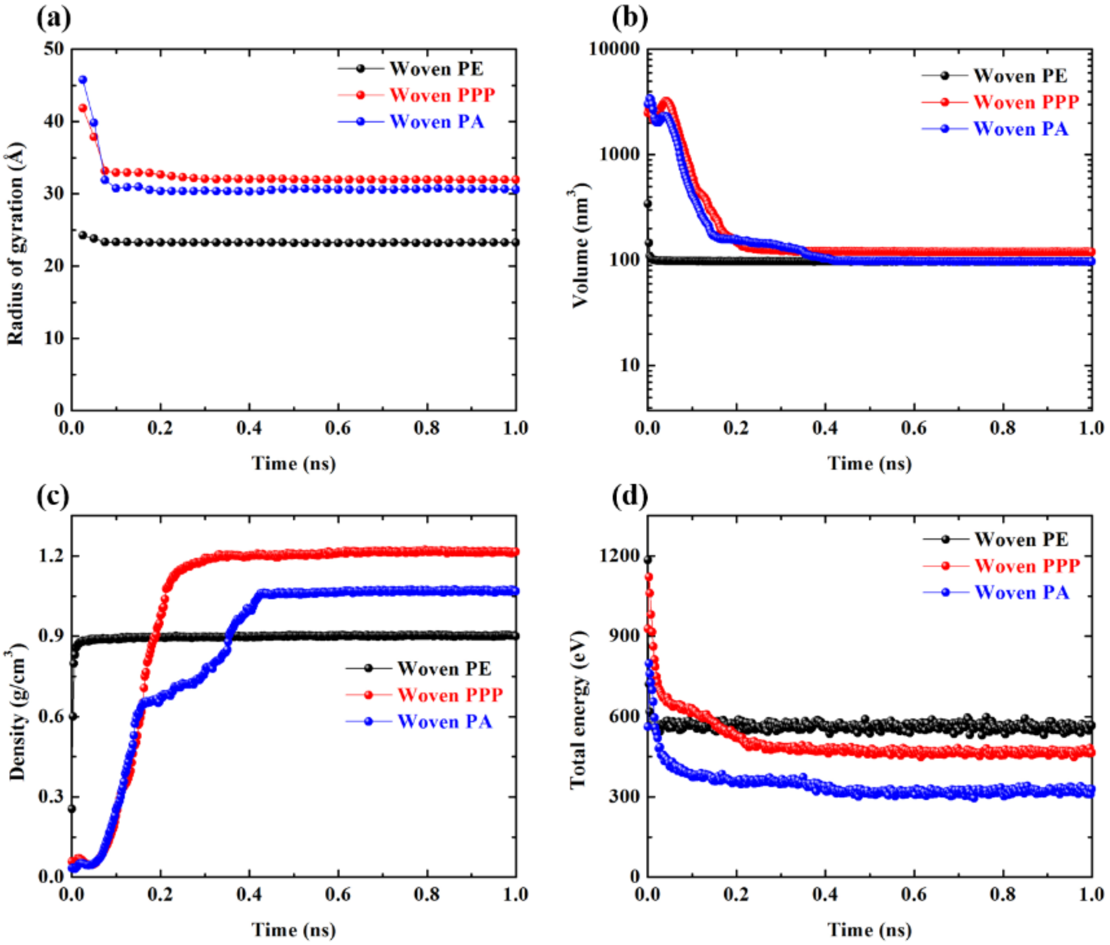

Figure S1. Typical physical property evolution of woven PE, woven PPP, and woven PA in the isothermal–isobaric (NPT) ensemble. (**a**) Radius of gyration evolution of woven PE, woven PPP, and woven PA in the NPT ensemble; (**b**) Volume evolution of woven PE, woven PPP, and woven PA in the NPT ensemble; (**c**) Density evolution of woven PE, woven PPP, and woven PA in the NPT ensemble; (**d**) Total energy evolution of woven PE, woven PPP, and woven PA in the NPT ensemble.

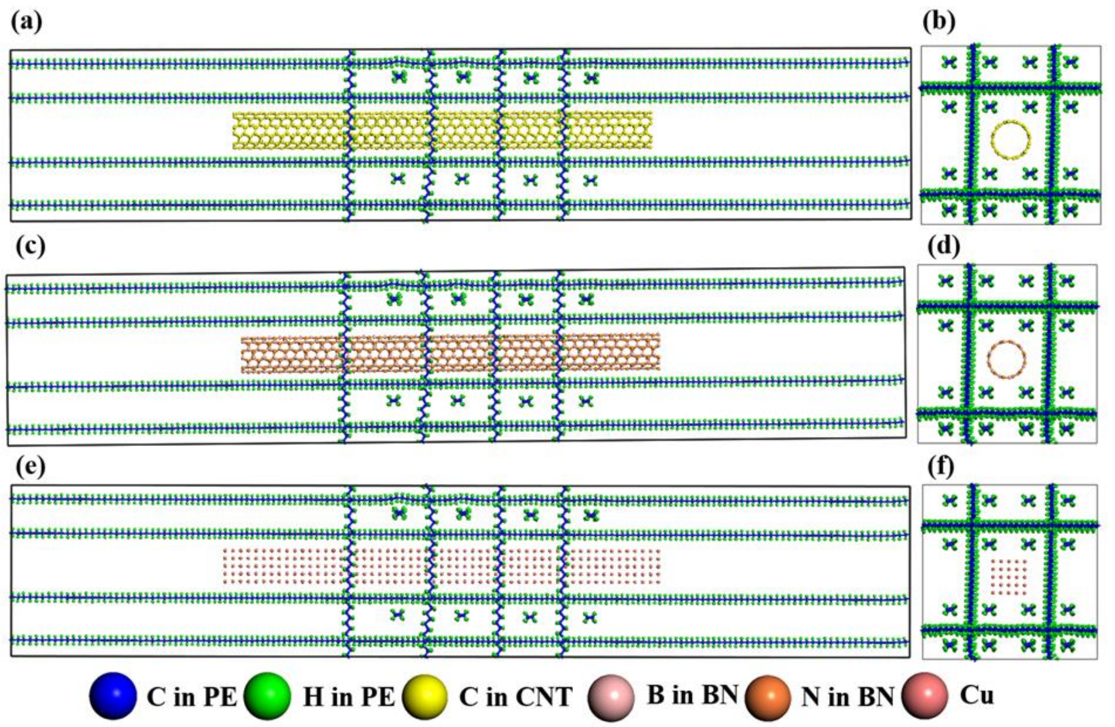

Figure S2. The pristine structure of PE-CNT, PE-BN, and PE-Cu. (**a**) Main view of PE-CNT; (**b**) Lateral view of PE-CNT; (**c**) Main view of PE-BN; (**d**) Lateral view of PE-BN; (e) Main view of PE-Cu; (**f**) Lateral view of PE-Cu.

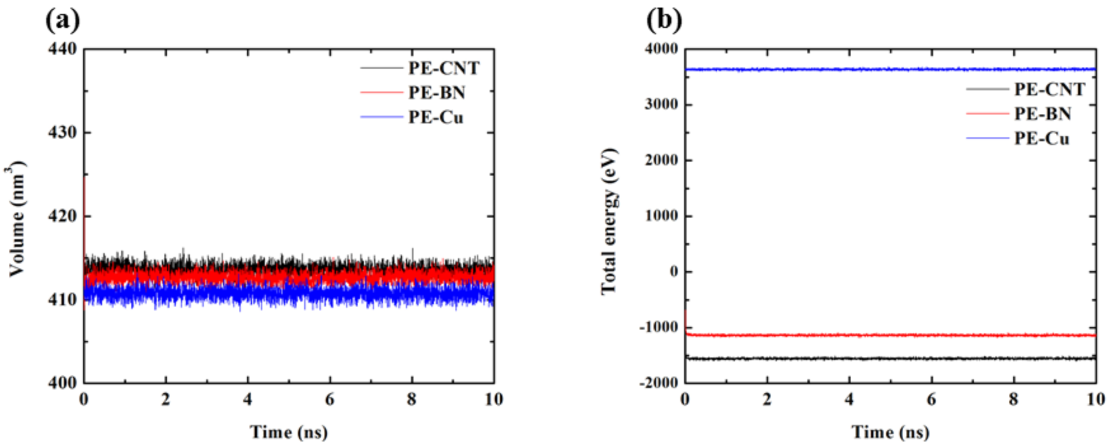

Figure S3. Typical physical property evolution of PE-CNT, PE-BN, and PE-Cu in the isothermal–isobaric (NPT) ensemble. (**a**) The volume evolution of PE-CNT, PE-BN, and PE-Cu in the NPT ensemble; (**b**) The total energy evolution of PE-CNT, PE-BN, and PE-Cu in the NPT ensemble.

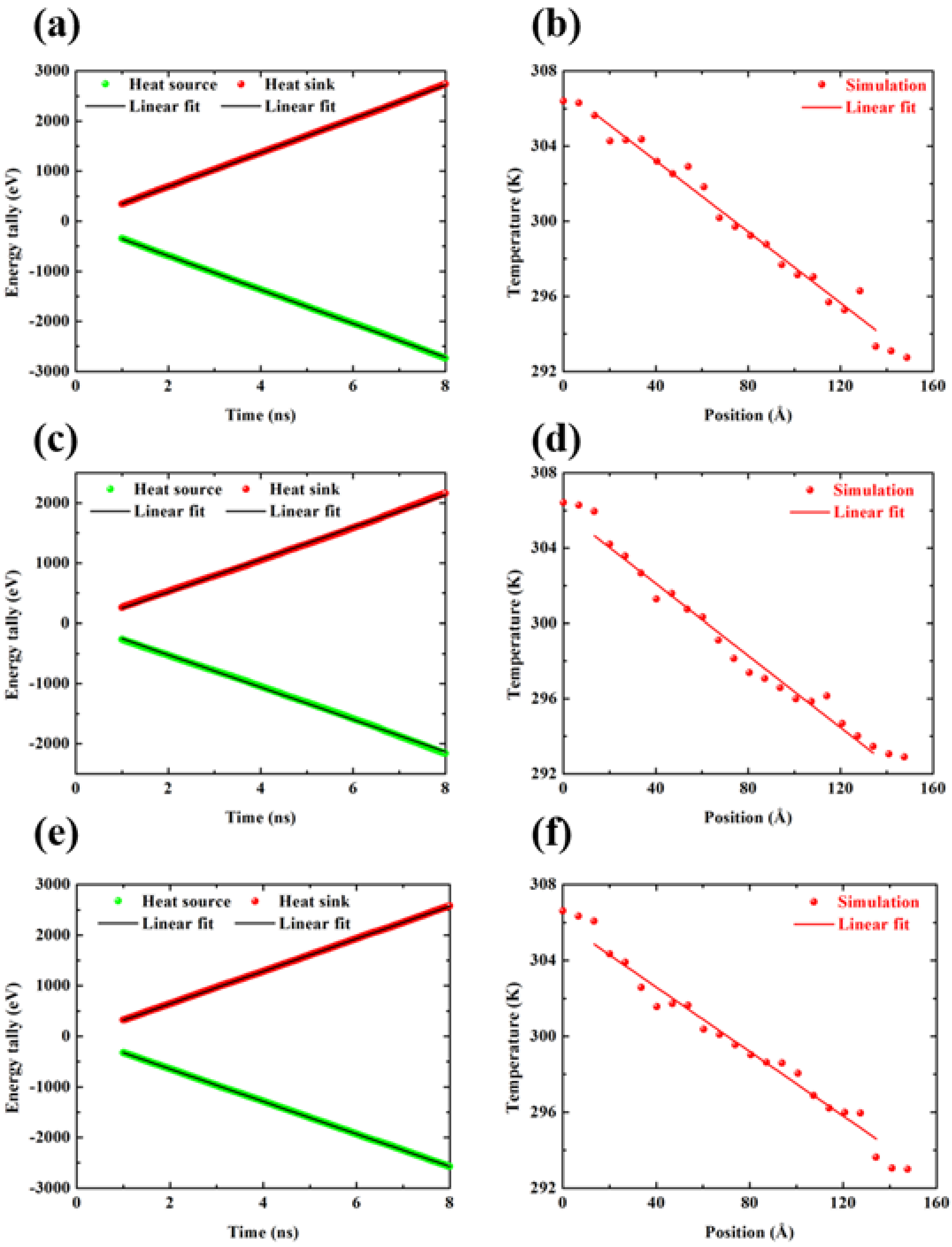

Figure S4. Steady state energy tally and temperature distribution in non-equilibrium molecular dynamics simulation for PE-CNT, PE-BN, and PE-Cu. (**a**) Steady state energy tally of PE-CNT; (**b**) Steady state temperature distribution of PE-CNT; (**c**) Steady state energy tally of PE-BN; (**d**) Steady state temperature distribution of PE-BN; (**e**) Steady state energy tally of PE-Cu; (**f**) Steady state temperature distribution of PE-Cu.
